# CD1-Restricted T Cells at the Crossroad of Innate and Adaptive Immunity

**DOI:** 10.1155/2016/2876275

**Published:** 2016-12-14

**Authors:** Catia S. Pereira, M. Fatima Macedo

**Affiliations:** ^1^Instituto de Investigação e Inovação em Saúde, Universidade do Porto, Porto, Portugal; ^2^Instituto de Biologia Molecular e Celular (IBMC), Universidade do Porto, Porto, Portugal; ^3^Departamento de Ciências Médicas, Universidade de Aveiro, Aveiro, Portugal

## Abstract

Lipid-specific T cells comprise a group of T cells that recognize lipids bound to the MHC class I-like CD1 molecules. There are four isoforms of CD1 that are expressed at the surface of antigen presenting cells and therefore capable of presenting lipid antigens: CD1a, CD1b, CD1c, and CD1d. Each one of these isoforms has distinct structural features and cellular localizations, which promotes binding to a broad range of different types of lipids. Lipid antigens originate from either self-tissues or foreign sources, such as bacteria, fungus, or plants and their recognition by CD1-restricted T cells has important implications in infection but also in cancer and autoimmunity. In this review, we describe the characteristics of CD1 molecules and CD1-restricted lipid-specific T cells, highlighting the innate-like and adaptive-like features of different CD1-restricted T cell subtypes.

## 1. Introduction

CD1-restricted T cells recognize lipid antigens bound to MHC class I-like CD1 molecules. The first paper describing CD1-restricted T cells was published in 1989, but the nature of the antigen presented was not identified [1]. The emergence of lipids as T cell antigens presented by CD1 molecules was only established 5 years later by the discovery of the antigenic properties of mycolic acid [2]. Nowadays, a variety of lipids, from both self- or non-self-origin, are known to bind CD1 molecules and to participate in lipid-specific T cell development and activation.

CD1-restricted T cells comprise specialized subtypes that participate in immune responses with innate-like and adaptive-like features. The relevance of these cells was described in the context of infection [3] and immune response against tumors [4]. Therefore, it has become pivotal to understand the properties of CD1 molecules, the mechanism of CD1-mediated lipid antigen presentation, and the biology of CD1-restricted T cells, to develop new strategies to control infection and cancer.

## 2. CD1 Molecules

Human CD1 molecules are encoded by 5 different genes localized to chromosome 1. These genes encode 5 different CD1 isoforms: CD1a–CD1e. The functional CD1 molecules are heterodimers composed by association of CD1 with *β*2-microglobulin. Based on sequence homology, CD1 isoforms can be classified into three groups. Group I is composed by CD1a, CD1b, and CD1c isoforms, group II by CD1d, and group III by CD1e.

### 2.1. Expression

Group I CD1 molecules are almost only expressed on thymocytes and dendritic cells (DCs) and are present in humans but not in mice or rats. CD1a is also expressed on Langerhans cells and CD1c in a subset of B cells [5]. CD1d has a wide expression pattern and is present in both hematopoietic and nonhematopoietic derived cells. CD1d is highly expressed on cortical thymocytes, but it gets downregulated in medullary thymocytes. In peripheral blood, B cells, monocytes, DCs, and activated T cells express CD1d. CD1d is also expressed in the gut, liver, bile duct epithelium, pancreas, kidney, endometrium, testis, epididymis, conjunctiva, breast, and skin [5]. In the human gut, intestinal epithelial cells express and present antigens by CD1d [5]. More recently, adipocytes were also found to express CD1d and a role in lipid antigen presentation has been suggested [6, 7]. CD1e is expressed on DCs but does not function as an antigen presenting molecule, since it is not present at the plasma membrane. This molecule functions as a lipid transfer protein (LTP) [8].

### 2.2. Structural Features

**Figure 1:**
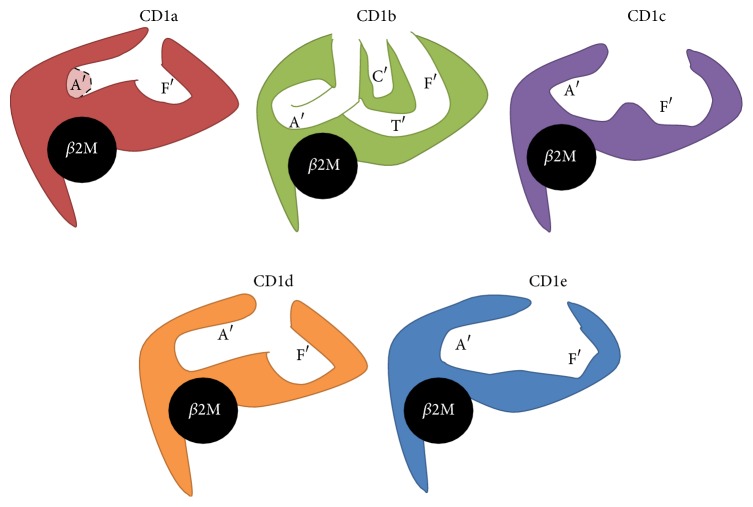
Schematic representation of the binding pockets from the different CD1 molecules (cross-sectional view). Dashed light colored area in CD1a represents the terminus of the A′ pocket. *β*2M: *β*2-microglobulin.

CD1 shares many structural features with MHC class I molecules. All CD1 isoforms are composed by a heavy chain that contains three extracellular domains (*α*1, *α*2, and *α*3), a transmembrane domain, and an intracellular tail. The *α*1 and *α*2 extracellular domains are composed by two antiparallel *α*-helices on the top of 6 *β*-strands. These are supported by the *α*3 domain that interacts with *β*2-microglobulin (the light chain) creating a heterodimer [9]. The striking difference between CD1 and MHC class I molecules relies on the antigen binding pockets. Contrary to MHC class I, CD1 pockets are lined by hydrophobic residues that interact with the hydrophobic part of the lipids while leaving the polar moieties exposed for TCR recognition [9]. The size, shape, and number of the pockets vary between CD1 isoforms, allowing the accommodation of lipids with variable fatty acid chain length (Figure 1) [10].

Similarly to MHC class I, CD1 molecules possess two deep pockets: A′ and F′. CD1b has two additional pockets, C′ and T′ that allow the binding of lipids with larger hydrophobic chains [11]. CD1a has the smallest binding groove and, contrarily to what is observed in the other CD1 isoforms, its A′ pocket is not directly connected to the other pockets, but instead it abruptly ends deep in the binding groove, functioning as a “molecular ruler” that prevents the binding of long hydrophobic chains (Figure 1) [12]. The F′ pocket is more permissive and allows binding of lipopeptides [13]. CD1a also has a semi-open conformation that facilitates the loading of lipids at neutral pH and without the action of LTP [12, 14]. CD1b has the larger binding site, composed of four pockets, three of which are interconnected to form a large A′T′F′ super channel. This characteristic confers CD1b the unique ability to bind long-chain mycolyl lipids [15]. The acidic pH of the lysosomes allows relaxation of CD1b, which improves lipid binding [16]. Similarly to CD1a, CD1c has an F′ pocket that is permissive to lipopeptide binding and usually associates with antigens that only have one alkyl chain, suggesting that the A′ pocket might be occupied by spacer lipids that stabilize CD1c structure [17]. CD1d was crystallized in complex with several lipids [18–21]. In all glycosphingolipids containing a ceramide backbone, the sphingosine chain binds the F′ pocket while the fatty acid occupies the A′ pocket, exposing the sugar head to the TCR. Despite its inability to present lipid antigens, the CD1e structure also contains A′ and F′ pockets, although they are not clearly separated, thus creating a larger groove [22]. Also, CD1e has a solvent exposed groove. These two characteristics together allow quick binding and release of different types of lipids, compatible with CD1e function of LTP [22].

### 2.3. Synthesis and Trafficking

After translation, CD1 molecules initiate their maturation process in the endoplasmic reticulum (ER). In the ER, glycosylation allows the binding of calnexin, calreticulin, and thiol oxidoreductase ERp57 that promote correct folding and assembly with *β*2-microglobulin [23]. Another ER protein with a pivotal role in CD1 assembly is microsomal triglyceride transfer protein (MTP). Absence of MTP results in severe defects in lipid antigen presentation by group I and group II CD1 isoforms [24–26]. The analysis of soluble CD1 molecules revealed that, during assembly, they are associated with different lipids rather than having empty pockets. Thus, it was suggested that MTP could load ER lipids into these pockets, stabilizing the molecules [26, 27]. However, another report showed that MTP absence does not alter the biosynthesis, glycosylation maturation, or plasma membrane internalization of CD1 molecules, but it is important for the recycling from the lysosome to the cell surface, suggesting another function for MTP besides CD1 stabilization through lipid loading [28].

**Figure 2:**
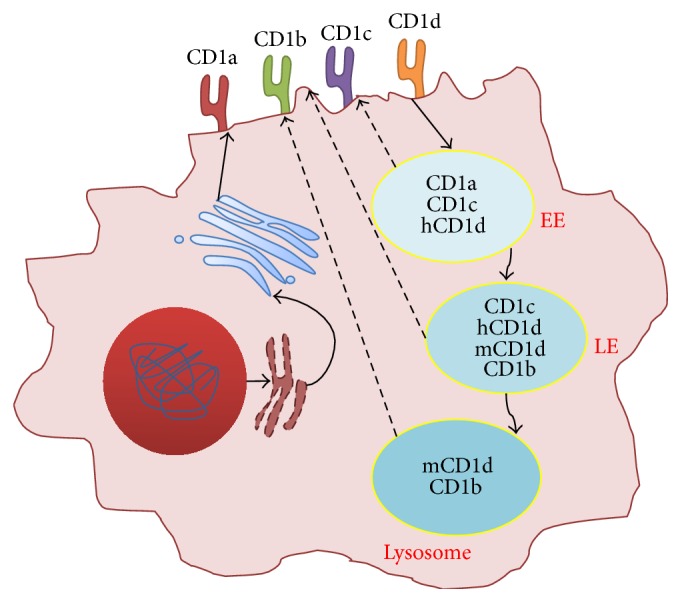
The cellular trafficking of CD1 molecules. After synthesis, CD1 molecules associate with *β*2-microglobulin in the endoplasmic reticulum. Then, they traffic to the trans-Golgi network, where they are glycosylated and follow to the plasma membrane (solid arrows). There, CD1 molecules are internalized by the endocytic pathway, where most of the loading occurs. The different CD1 isoforms localize in different endocytic compartments. The loaded CD1 molecules then traffic to the plasma membrane, where they activate T cells (dashed arrows). EE: early endosome; LE: late endosome; hCD1d: human CD1d; mCD1d: mouse CD1d.

CD1 molecules continue their maturation in the trans-Golgi network (Figure 2). The identification of some Golgi-synthesized lipids bound to CD1 suggests that they are loaded along the secretory pathway, after exiting the ER [29]. In the trans-Golgi network, CD1 molecules also complete their glycosylation process before being exposed at the cell surface. When in the plasma membrane, CD1 molecules are recycled through the endosomal route, where they encounter lipid antigens (Figure 2). Internalization of CD1b, CD1c, and CD1d is mediated by interaction of the cytoplasmic tail with the adaptor protein complex- (AP-) 2, which sorts cargo proteins into clathrin-coated pits [30–33]. Contrarily, CD1a does not interact with AP-2 and is internalized using clathrin and dynamin-independent pathways [34]. After internalization into sorting endosomes, the different CD1 isoforms have distinct fates (Figure 2). CD1a and CD1c localize in the endocytic recycling compartment, which indicates that they follow the slow recycling pathway back to the plasma membrane. However, CD1c can also be found in late endosomes. CD1b and mouse CD1d (mCD1d) interact with AP-3, which sorts these molecules to the late endosomes and lysosomes. Curiously, human CD1d does not interact with AP-3 and can be found in late endosomes [35]. Studies with mCD1d lacking the cytoplasmic tail (and therefore not internalized for recycling) revealed the presence of mCD1d molecules in lysosomes, suggesting the existence of an alternative pathway that directly sorts mCD1d to lysosomes [36]. This was explained by the association of mCD1d with the invariant chain (Ii) and MHC class II in the ER, which directly sends mCD1d to MHC class II compartments or lysosomes [37]. Later, Ii was also shown to associate with CD1a, suggesting that this might be applicable to all CD1 isoforms [38]. After reaching the endocytic compartments, CD1 molecules exchange the nonimmunogenic lipids acquired during assembly with antigenic lipids, with the help of several LTP. The mechanisms responsible for the targeting of CD1 molecules from the lysosome to the plasma membrane are not well understood, but it is known that localization of these molecules in lipid rafts improves antigen presentation [39]. Recently, it was shown that lysosomal pH had an influence on CD1d localization at the plasma membrane [40].

### 2.4. CD1-Binding Lipids

Lipid antigens include mostly phospholipids and sphingolipids (Table 1). Interestingly, sphingolipids are the only lipids shown to be presented by all CD1 isoforms, so far. However, a variety of lipid classes were shown to bind some CD1 isoforms and activate CD1-restricted T cells (Table 1). Curiously, some antigens can be presented by more than one CD1 isoform. The most striking example is sulfatide that has the unique property of binding and activating T cells restricted to all CD1 isoforms [14].

Not all CD1-binding lipids are immunogenic. Another important group of CD1 binding lipids is spacer lipids. CD1 isoforms typically bind lipids with hydrophobic chains that match the size of the binding groove, suggesting a 1 : 1 stoichiometry. However, CD1b was found to be associated with rather small lipids that do not fully occupy the binding pocket. This raised the question of whether CD1b was able to bind two lipids simultaneously. Crystallography analysis of CD1b structure and lipidomics analysis of CD1b eluted lipids identified, besides the antigenic lipid, several spacer lipids that stabilize the CD1b molecule and that rearranged upon binding to allow antigen recognition [68]. Evidence from crystallographic studies also suggests the presence of spacer lipids in CD1a, CD1c, and CD1d [19, 66, 69].

Among nonimmunogenic CD1-binding lipids, we can also find molecules with inhibitory properties. The glycosphingolipid globotriaosylceramide was shown to bind CD1d and inhibit the activation of a subset of CD1d-restricted T cells, the invariant Natural Killer T (iNKT) cells [70]. The inhibition is achieved through a direct competition between globotriaosylceramide and iNKT cell antigens for CD1d binding. It is possible that this inhibitory characteristic is shared by other CD1 binding lipids that are not recognized by a TCR, thus representing an important mechanism for controlling the activation of lipid-specific T cells.

### 2.5. Lipid Loading on CD1 Molecules

Lipids are hydrophobic and therefore need assistance for transport, uptake, and processing. This role is played by LTPs. In the bloodstream, lipids travel in very low density or high density lipoprotein particles or associated with some monomeric proteins [13]. The uptake of the lipid antigens by the cells occurs by interaction with cellular receptors such as low density lipoprotein receptors and scavenger receptors. The receptor usage seems to be dependent on the type of cell and influenced by the lipid structure [13, 71, 72]. Lipid structure also influences intracellular trafficking. While lipid antigens with short unsaturated alkyl chains localize in the endocytic recycling compartment, lipids with long saturated tails travel to the late endocytic compartments [13]. This difference in trafficking allows the encounter of the different CD1 isoforms with their preferred ligands.

In endocytic compartments, specialized LTPs assist lipid binding to CD1. Although some self-lipids are loaded into CD1 during folding in the ER, exogenous lipids need to be loaded from membranes or lipid-protein complexes, once internalized. The lysosomal proteins that facilitate this process include saposins, GM2 activator protein, Niemann-Pick C2 (NPC2) protein, and CD1e [8, 73–81]. Saposins are a group of 4 proteins that arise due to cleavage of a common precursor: prosaposin. They were shown to be important for endogenous and exogenous lipid removal and loading into mouse and human CD1d, both in the steady-state and during infection [75–77, 81]. Saposin B greatly improves human CD1d-mediated lipid antigen presentation, but saposins A and C were also shown to efficiently perform lipid exchange in mCD1d molecules [75, 76, 81]. Saposin C binds both CD1b and CD1c, facilitating lipid loading in these molecules [73, 74]. Importantly, this function is restricted to lipid exchange, meaning that saposins are not capable of removing lipids from CD1 if they cannot be replaced by another. GM2 activator protein is a cofactor for *β*-hexosaminidase A but it also removes mCD1d bound-lipids, without the need of binding other lipids [81]. A similar function was found for the NPC2 protein [78]. CD1e was described as an isoform incapable of presenting lipid antigens, due to its absence from the plasma membrane. However, the endosomal localization and the similarities in the binding pocket shared by the different CD1 isoforms suggested that CD1e binds lipid antigens. In 2005, the role of CD1e in lipid antigen processing was demonstrated by the identification of CD1e as a cofactor for *α*-mannosidase, a lysosomal enzyme that in the presence of CD1e degrades complex nonimmunogenic mycobacterial lipids to antigenic forms [8]. Importantly, CD1e promotes the loading and unloading of lipids into CD1d [80] and also influences lipid presentation by CD1b and CD1c [80].

Besides LTP, CD1 lipid exchange in endosomal compartments is also facilitated by the low pH that induces relaxation of the CD1 structure, promoting a more dynamic binding and dissociation of lipids [16, 71].

## 3. CD1-Restricted T Cells

CD1-restricted T cells can be classified as restricted to group I CD1 molecules or to CD1d. CD1d-restricted T cells are also designated Natural Killer T (NKT) cells, because most of these cells share surface markers of NK and T cells. NKT cells are further divided into two subsets. Type I NKT cells, or iNKT cells, are characterized by the expression of a semi-invariant TCR (V*α*24J*α*18V*β*11 in humans and V*α*14J*α*18 paired with a limited repertoire of V*β* chains in mice) and by the recognition of the lipid antigen *α*-galactosylceramide (*α*-GalCer) [82]. Type II NKT cells recognize a variety of lipid antigens and express variable TCRs, although with a bias towards some V*α* and V*β* chains [82].

Group I CD1-restricted T cells are polyclonal and probably undergo clonal expansion at the periphery, after antigen encounter. This results in a delayed effector response, consistent with an adaptive-like immune response, similar to what is observed for MHC-restricted T cells [4]. iNKT cells differ from most T cells due to their innate-like functions. After expansion and maturation in the thymus, iNKT cells are capable of responding to innate signals, such as cytokine stimulation, within hours. However, they also respond to TCR engagement by specific antigens, thus standing in the middle of the innate and adaptive immune response.

### 3.1. Adaptive-Like Group I CD1-Restricted T Cells

To date, there is no specific method to identify all lipid-specific group I CD1-restricted T cells. However, studies analyzing self-reactive group I CD1-restricted T cells described a high frequency of these cells, similar to what is observed for autoreactive conventional T cells [83]. Furthermore, autoreactive group I CD1-restricted T cells are present in both umbilical cord blood and peripheral blood at similar frequencies [83]. They express mainly the marker CD45RA, but a decrease of CD45RA-positive cells is seen in peripheral blood when compared with umbilical cord blood, consistent with an adaptive-like phenotype [83]. Also in accordance with the adaptive-like phenotype of these cells, the presence of* Mycobacterium tuberculosis*-specific CD1b-restricted T cells is dependent on previous contact with* M. tuberculosis *[84].

Upon activation, group I CD1-restricted T cells present a Th0 or Th1 phenotype, producing large amounts of IFN-*γ* and TFN-*α*. They can also display cytotoxic activity and induce the lysis of intracellular mycobacteria [83–85].

CD1a-restricted T cells are among the most frequent self-reactive CD1-restricted T cells in peripheral blood [83, 86]. Moreover, they are common in the skin. Skin CD1a-restricted T cells become activated when in contact with CD1a expressed by Langerhans cells. Upon activation, they produce IFN-*γ*, IL-2, and IL-22, a cytokine with suspected roles in skin immunity [86]. CD1a-restricted T cells are unique in the way that their TCR can directly recognize the CD1a molecule without corecognition of a lipid antigen [48]. Self-ligands for CD1a can be either permissive, such as lysophosphatidylcholine that allows activation of autoreactive T cells as it allows the contact of the CD1a with the TCR, or nonpermissive, such as sphingomyelin that disrupts the TCR-CD1a contact zone and in this way does not allow activation of CD1a-restricted T cells [48]. Nevertheless, some CD1a-restricted T cell clones were shown to recognize antigens protruding out of the CD1a pocket, such as sulfatide [12, 14], indicating that some TCRs require a lipid antigen for recognition.

The number of CD1b-restricted self-reactive T cells in blood is very low [83, 86]. CD1b-restricted T cells seem to be especially important in mycobacterial immunity [84, 87–89]. More recently, lipids from* Staphylococcus aureus*,* Brucella melitensis*, and* Salmonella *Typhimurium were shown to activate CD1b-restricted T cells [44]. Interestingly, these cells also displayed autoreactivity, indicating that bacteria and mammalian cells share CD1b antigens.

The frequency of CD1c-autoreactive T cells is not consensual in the literature [83, 86], with one study reporting a very low frequency [86] and a second study reporting an intermediated frequency between the high frequent CD1a autoreactive T cells and the low frequent CD1b and CD1d autoreactive T cells [83]. Although CD1c is widely expressed in DCs and B cells from peripheral blood, only sulfatide and mLPA were identified as self-antigens presented by CD1c (Table 1) [14, 59]. Similarly to what was observed for other CD1-restricted T cells, mycobacterial lipids induce CD1c-dependent T cell responses (Table 1) [66].

### 3.2. Innate-Like CD1-Restricted T Cells: iNKT Cells

iNKT cells are easily identified by staining with CD1d tetramers loaded with *α*-GalCer or with antibodies against the semi-invariant TCR. Thus, these are the most studied lipid-specific T cells. iNKT cell frequency varies between mice and humans. In mice, iNKT cells are more frequent in the liver and adipose tissue and are present at a lower percentage in thymus, spleen, bone marrow, peripheral blood, and lymph nodes. In humans, iNKT cells are more frequent in the adipose tissue, followed by the liver, and appear at lower percentages in spleen, peripheral blood, lymph nodes, bone marrow, and thymus [90].

An important feature of iNKT cells is related to their ability to quickly produce large amounts of cytokines upon stimulation, either by a TCR-dependent or independent manner [91]. This innate-like phenotype of iNKT cells is further demonstrated by the expression of CD45RO in humans and CD44 in mice and the early activation marker CD69 [82, 92]. Furthermore, iNKT cells display high autoreactivity. To date, the mechanisms that allow the control of iNKT cell autoreactivity are not completely understood. However, it has been shown that some self-lipids are capable of inhibiting iNKT cell activation and therefore may function as limiters of iNKT cell activation [70, 93].

The development of iNKT cells starts in the thymus by interactions of CD1d loaded with self-antigens, expressed in double-positive (DP) thymocytes, with DP thymocytes expressing the semi-invariant TCR [13]. This interaction ultimately leads to the expression of the transcription factor PLZF and iNKT cell maturation. In mice, iNKT cells express different types of transcription factors that drive them to NKT1, NKT2, or NKT17 subsets (Table 2).

NKT1 cells express mainly IFN-*γ*, high levels of T-bet, and low levels of GATA3. They are also characterized by NK1.1 expression, absence of IL-17RB, and dependence on IL-15 [94]. During differentiation, these cells downregulate PLZF [95].

NKT2 cells produce mainly IL-4 and are characterized by the expression of the transcription factor GATA-3 [94, 95]. They are localized mainly in the lung and are more frequent in BALB/c mice. Contrary to NKT1 cells, NKT2 cells are dependent on IL-17RB expression for development and express high levels of PLZF [95]. In humans, the functional properties of CD4+ iNKT cells are highly associated with the NKT2 phenotype [96–98].

The NKT17 subset is characterized by the preferential production of IL-17 and IL-22, instead of IL-4 and IFN-*γ* [94]. They were identified within NK1.1− CD4− cells and are mainly present in the lung, lymph nodes, and skin [99, 100]. Recently, they were shown to express syndecan-1 [101]. Despite the fact that some IL-17 producing cells are committed to this fate in the thymus, iNKT cells can also acquire this ability in the periphery, under certain conditions [102]. At the transcriptional level, the development of NKT17 cells is repressed by ThPOK and driven by ROR*γ*t expression [103–105]. E protein was also shown to be important to drive subset commitment. Increased expression of this protein leads to a reduction in NKT1 cells with an increase in NKT2 and NKT17 cells [106].

So far, the existence of these subsets in humans was not clarified. Thus, in humans, iNKT cell subsets are still defined based on the expression of cell surface molecules (such as CD4 and CD8) and cytokine production. Contrary to what is observed in mice, iNKT cells in humans can express only CD4, only CD8, or none of the molecules. Importantly, CD4 and CD8 expression defines functionally distinct subsets. CD4− iNKT cells (which include both CD8+ and double negative cells) are characterized by a Th0 phenotype, while CD4+ iNKT cells tend to produce larger amounts of Th2 cytokines [96–98, 107]. Among CD4− iNKT cells, those expressing CD8 present a Th1 bias, producing larger amounts of IFN-*γ* and almost no IL-4, when compared to double negative cells [98]. They also display the highest cytotoxic activity [98]. Another subset is characterized by cells producing IL-17 that arise in response to proinflammatory conditions and express CD161 [108]. It is therefore necessary to analyze the different iNKT cell subsets in pathology, since their impact in disease may be different. Indeed, alterations in iNKT cell CD4+/CD4− subsets were described in Fabry disease, a lysosomal storage disease characterized by accumulation of glycosphingolipids, despite the fact that a normal percentage of total iNKT cells was observed in the peripheral blood of patients [109–111].

### 3.3. Type II NKT Cells: A Mixed Population of Innate-Like and Adaptive-Like T Cells

Type II NKT cells are the most frequent CD1d-restricted T cells in humans but represent the minority in mice [112]. Contrary to iNKT cells, type II NKT cells express diverse TCRs and respond to a variety of lipid antigens, of either self- or non-self-origin (Table 1). Thus, identifying the whole population of type II NKT cells is currently a challenge. Initially, the comparison of MHC-deficient mice (lacking conventional T cells) with MHC/CD1d double knockouts described a population of CD4+ non-*α*-GalCer reactive T cells that displayed an effector memory phenotype and bias towards some autoreactive TCRs [113, 114].

More recently, using 4get mice (in which cells expressing IL-4 are GFP+) type II NKT cells were shown to constitutively express IL-4 [115]. Thus, these mice were crossed with J*α*18^−/−^, to obtain a model in which type II NKT cells are identified by GFP expression [115]. A polyclonal population that shares some developmental traits with iNKT cells was characterized. Deficiency of SAP and PLZF compromises the development of iNKT cells but also leads to decreases in type II NKT cell percentage [115]. Phenotypically, these polyclonal type II NKT cells are very similar to iNKT cells. They are characterized by an activated memory state, as determined by CD69 and CD44 expression. Regarding coreceptor expression, they can express only CD4 or neither CD4 nor CD8 [115]. However, they are distinct from iNKT cells when considering cytokine production. They produce less IL-4 and less IFN-*γ*, but similar levels of IL-13 and GM-CSF [115]. Although polyclonal, type II NKT cells showed a bias towards the usage of TCR V*β* 8.1/8.2 chains [115].

A different approach for the characterization of type II NKT cells relies in the use of CD1d tetramers loaded with lipid antigens. Staining of human PBMCs with sulfatide-loaded CD1d tetramers revealed that most of sulfatide-reactive NKT cells possess *γδ* TCRs, expressing the V*δ*1 segment [116]. Another report that characterized *β*-glucosylceramide and *β*-glucosylsphingosine-specific type II NKT cells showed that these cells could express CD4 or CD8 [55]. Furthermore, these cells can convert to a T follicular-helper phenotype upon injection of antigen and induce antibody production, germinal center formation, and the differentiation of B cells in plasmablasts, indicating a role in help to B cells, as previously described for iNKT cells [55]. Importantly, the *β*-glucosylceramide and *β*-glucosylsphingosine-specific type II NKT cells identified in this study mainly expressed CD45RA, consistent with a naïve phenotype, instead of the effector memory phenotype previously described in mice [55].

Altogether, these studies suggest that type II NKT cells represent a heterogeneous group of CD1d-restricted T cells, with cells that display an innate-like response similar to iNKT cells, but also with other cells, displaying adaptive-like immune functions. The relative contribution of the innate-like and adaptive-like cells for the overall group of type II NKT cells is still unclear.

## 4. Concluding Remarks

Lipid-specific CD1-restricted T cells comprise an important part of the immune system. However, the existent studies so far were not able to completely characterize and unequivocally include CD1-restricted T cells in the innate or adaptive immune responses. Instead, they stand at the crossroad of these responses and may have an important role in bridging the adaptive and the innate arms of the immune system. A complete characterization of lipid-specific CD1-restricted T cells is hampered by the lack of specific markers to identify the different CD1-restricted T cell populations. Hence, most of the information available on these cells arose from the study of individual T cell clones. Although valuable, this information may not be representative of the* in vivo* dynamics. In the past few years, great progresses were made in this field, mainly due to the development of CD1 tetramers loaded with lipid antigens. Using CD1 tetramers, it is possible to analyze lipid-specific CD1-restricted T cells* ex vivo* and to phenotypically and functionally characterize them. Lipid antigens were shown to be present in cancer cells and infectious agents, and therefore the complete knowledge of these cells is important to develop new strategies against cancer and infectious diseases.

## Figures and Tables

**Table 1:** Naturally occurring antigens for CD1-restricted T cells.

Class	Lipid	Origin	CD1	References
Phospholipids	PE	Cypress; self	CD1a; mCD1d	[41, 42]
	PC	Cypress; self	CD1a; hCD1d; CD1c	[41, 43]
	PG	*M. tuberculosis; L. monocytogenes; E. coli; C. glutamicum * Self	CD1b; mCD1d	[42, 44–46]
	PI	*M. tuberculosis;* self	mCD1d	[42, 46]
	Cardiolipin	Self	mCD1d	[18]
	DPG	*M. tuberculosis; L. monocytogenes *	mCD1d	[45, 46]
	Lyso-PE	Self	m/hCD1d	[47]
	Lyso-PC	Self	m/hCD1d; CD1a	[47–49]

Sphingolipids	Lyso-Sph	Self	hCD1d	[49]

Glycosphingolipids	Sulfatide	Self	CD1a; CD1b; CD1c; m/hCD1d	[14, 50]
	Lysosulfatide	Self	mCD1d	[50]
	GM1	Self	CD1b	[51]
	GD3	Self	mCD1d	[52]
	*α*-GalCer	Self	m/hCD1d	[53]
	*β*-GlcCer	Self	mCD1d	[54]
	Lyso-GalCer	Self	mCD1d	[54]
	*β*-GlcSph	Self	m/hCD1d	[54, 55]
	*β*-GalCer	Self	mCD1d	[54]
	iGb3	Self	mCD1d	[56]
	GSL-1	*Sphingomonas *spp.	m/hCD1d	[57]

Plasmalogens	pLPE	Self	m/hCD1d	[58]
	mLPA	Self	CD1c	[59]
	eLPA	Self	mCD1d	[58]

Oils	Triacylglyceride	Self	CD1a	[60]

Terpenes	Squalene	Self	CD1a	[60]

Diacylglycerolipids	GalDAG	*B. burgdorferi*	m/hCD1d	[61]

Mycolates	GMM	*M. tuberculosis*	CD1b	[62]
	Mycolic acid	*M. tuberculosis*	CD1b	[2]

Lipoglycans	PIM	*M. tuberculosis*	CD1b	[63]
	LAM	*M. tuberculosis*	CD1b	[63]
	LPG	*L. donovani*	mCD1d	[64]

Lipopeptides	Dideoximycobactin	*M. tuberculosis*	CD1a	[65]

Mycoketides	MPM	*M. tuberculosis*	CD1c	[66, 67]
	PM	*M. tuberculosis*	CD1c	[66]

PE: phosphoethanolamine; PC: phosphatidylcholine; PG: phosphatidylglycerol; PI: phosphatidylinositol; DPG: diphosphatidylglycerol; Sph: sphingomyelin; GalCer: galactosylceramide; GlcCer: glucosylceramide; GlcSph: glucosylsphingosine; iGb3: isoglobotriaosylceramide; GSL-1: glycosphingolipid 1; pLPE: lysophosphatidylethanolamine; mLPA: methyl-lysophosphatidic acid; eLPA: lysophosphatidic acid; GalDag: galactosyldiacylglycerol; GMM: glucose monomycolate; PIM: phosphatidylinositol mannose; LAM: lipoarabinomannan; LPG: lipophosphoglycan; MPM: mannosyl phosphomycoketide; PM: phosphomycoketide.

**Table 2:** Main iNKT cell subsets in mice: transcriptional programs, surface markers, cytokine production, and frequency.

	Transcriptional factors	Surface markers	Cytokine production	Frequency^*∗*^
NKT1	T-bet^hi^	NK1.1	IFN-*γ*	Most frequent subset
	GATA-3^lo^	IL-17RB^−^	IL-4	
	PLZF^−^	IL-15R*α* ^+^		
	Id2^+^	CD4^+/−^		

NKT2	T-bet^lo^	NK1.1^−^	IL-4	More common in the lung
	GATA-3^hi^	IL-17RB^+^	IL-13	
	PLZF^high^	CD4^+^		
	Id3^+^			

NKT17	ROR*γ*t^+^	NK1.1^−^	IL-17	Mainly present in the lung, lymph nodes, and skin
	PLZF^int^	IL-17RB^+^	IL-22	
		CD4^−^		

^*∗*^In C57BL/6 mice. hi: high; lo: low.
